# Biogenic Zinc oxide nanoparticles from *Celosia argentea*: toward improved antioxidant, antibacterial, and anticancer activities

**DOI:** 10.3389/fbioe.2023.1283898

**Published:** 2023-12-15

**Authors:** Rana Abdullah Alghamdi, Maryam Hassan Al-Zahrani, Lamaia R. Altarjami, Waleed Al Abdulmonem, Nadia Samir, Alaa Said, Ashjan A. Shami, W. S. Mohamed, Mohammed Ezzeldien

**Affiliations:** ^1^ Department of Chemistry, College of Sciences and Arts, King Abdulaziz University, Rabigh, Saudi Arabia; ^2^ Regenerative Medicine Unit, King Fahd Medical Research Centre, King Abdulaziz University, Jeddah, Saudi Arabia; ^3^ Biochemistry Department, Faculty of Science, King Abdulaziz University, Jeddah, Saudi Arabia; ^4^ Department of Pathology, College of Medicine, Qassim University, Buraidah, Saudi Arabia; ^5^ Zoology Department, Faculty of Science, South Valley University, Qena, Egypt; ^6^ Electronic and Nano Devises Lab, Faculty of Science, South Valley University, Qena, Egypt; ^7^ Department of clinical laboratory sciences, College of applied medical sciences, Taif University, Taif, Saudi Arabia; ^8^ Physics Department, College of Science, Jouf University, Al-Jouf, Sakaka, Saudi Arabia; ^9^ Physics Department, Faculty of Science, Sohag University, Sohag, Egypt; ^10^ Metallurgy and Material Science Tests Lab, Physics Department, Faculty of Science, South Valley University, Qena, Egypt

**Keywords:** *Celosia argentea*, ZnO NPs, antioxidant, antibacterial, anticancer

## Abstract

Biogenic Zinc oxide (ZnO) nanoparticles (NPs) were synthesized from *Celosia argentea* (*C. argentea*) plant extract. Structural analysis confirms the successful synthesis of biogenic zinc oxide NPs from *C*. *argentea* extract. The biogenic ZnO NPs have an average particle size of 21.55 ± 4.73 nm, a semispherical shape, and a specific surface area of about 50 m^2^/g. The biogenic ZnO NPs have a powerful radical scavenging activity (Ic_50_ = 91.24 mg/ml) comparable to ascorbic acid (ASC) as a standard (Ic_50_ = 14.37 mg/ml). The antibacterial efficacy was tested against gram-positive and gram-negative bacteria using an agar disc diffusion method. Gram-positive strains with biogenic ZnO NPs have a greater bactericidal impact than gram-negative strains in a concentration-dependent manner. Anticancer activity against Liver hepatocellular cells (HepG2) and Human umbilical vein endothelial cells (HUVEC) was evaluated using a [3-(4,5-dimethylthiazol-2-yl)-2,5diphenyl tetrazolium bromide] (MTT) assay. The results reflect the concentration-dependent cytotoxic effect of biogenic ZnO NPs against HepG2 cells even at low concentrations (Ic_50_ = 49.45 μg/ml) compared with doxorubicin (Ic_50_ = 14.67 μg/ml) and *C. argentea* extract (Ic_50_ = 112.24 μg/ml). The cell cycle and gene expression were analyzed to determine the potential anticancer mechanism. The flow cytometric analysis of the cell cycle revealed that biogenic ZnO NPs induce oxidative stress that activates the apoptotic genes NF-κB, CY-C, and P53, leading to cell death. The *Celosia argentea* improved the antioxidant, antibacterial, and anticancer activities of ZnO NPs without altering their structural properties. The effect of green synthesis on the bioactivity of biogenic ZnO NPs *in vivo* is recommended for future work.

## 1 Introduction

The global demand to develop new pharmaceuticals has increased to overcome existing drugs’ limitations and side effects. Multidrug resistance is an example of complicated health issues raised by the uncontrolled use of antibiotics. Another example is the use of anticancer drugs such as doxorubicin, dactinomycin, cisplatin, and tamoxifen, which are associated with severe side effects and have limited effect of action. Therefore, after the nanotechnology revolution, scientific committees focused on developing drugs with adoptive bioactivity for enhanced antibacterial, antioxidant, anticancer, and anti-inflammatory activities. A dozen research articles were published on controlling NPs properties to be suitable in the biomedical field to diagnose and treat different pathogens.

A key factor in NPs characteristics and functionalization is the synthesis method. Three main methods were reported for NPs synthesis: physical, chemical, and biological. The physical methods include mechanical milling and physical vapor deposition (Khan et al., 2019a). At the same time, chemical methods use chemical reactions to form ions to reconstruct NPs, such as chemical vapor deposition, chemical precipitation, and sol-gel processes (Wang et al., 2021). On the other hand, the biological methods use the biomolecules in microorganisms as a reducing and capping agent (Ansari et al., 2023; Khan et al., 2023). Biological and eco-friendly processes have attracted attention due to their low cost, reduced toxicity, and design flexibility (Das and Chatterjee, 2019). Utilizing microorganisms and plant extracts as reducing and capping agents facilitates the formation of NPs (Sorbiun et al., 2018). These biomolecules control particle size and shape, functionalize the surface of NPs, and enhance their biocompatibility and bioactivity (Khan et al., 2019b). The future of green synthesis of NPs is expanding to include several microorganisms, including viruses, bacteria, algae, and yeast (Makarov et al., 2014; Patil and Chandrasekaran, 2020). Plant extract is an easy, scalable, flexible, and eco-friendly method (Baker et al., 2013). The technique provides NPs functionalized with different phytochemicals, which can enhance the biocompatibility and bioactivity of NPs to serve efficiently in many biomedical applications (Makarov et al., 2014).

Zinc oxide is a very distinguished semiconductor in the biomedical field. The antibacterial, anti-inflammatory, antifungal, antiviral, and anticancer activities of ZnO NPs were reported by many researchers (Kang et al., 2013; Bisht and Rayamajhi, 2016; Rajashekara, 2020). Green synthesized ZnO NPs from different plant extracts were confirmed to have enhanced bioactivity. Biogenic ZnO NPs were synthesized from the extract of *M. pulegium* and *Peltophorum pterocarpum* with comparable antibacterial activity against gram-positive and gram negative bacterial strains (Pai et al., 2019; Rad et al., 2019). *M. indica* and *Tabernaemontana divaricata* extracts were also used to produce ZnO NPs with free radical scavenging and anticancer activities (Raja et al., 2018; Rajeshkumar et al., 2018). The antioxidant activity of green ZnO NPs was attributed to the phytochemicals in the plant extract, such as phenolics and polyphenolic compounds (Ramesh et al., 2022). The higher activity to accept and donate hydrogen ions in the physiological media depends highly on its physiochemical properties, which can be tuned with the biomolecules in green synthesis. Moreover, the green synthesis can also modify the high surface-to-volume ratio, which offers more sites to generate ROS. ROS was reported to be the hidden soldier for the cytotoxic effect of ZnO NPs (Hamed et al., 2023). Additionally, ZnO NPs were approved to be more selective to highly proliferative cells, such as cancer cells, due to a higher proliferation rate and faster metabolic rate than low proliferative cells (normal cells) (Raffa et al., 2011; Alrokayan et al., 2012; Valdiglesias et al., 2013).

*C. argentea* is a medical plant belonging to the Amaranthaceae family (see Table 1) and commonly found in Africa and Asia (Thorat, 2018). It has many names; plumed cockscomb or silver cock’s comb. *C. argentea* is rich in many vital minerals such as Ca – 178.08 mg, P – 38.01 mg, K – 62.34 mg, Na – 35.25 mg, Mg – 39.64 mg, Fe – 15.25 mg, Zn – 7.25 mg, and Cu – 3.75 mg per 100 g of sample with a trace amount of Cr, Mn, Ni, and Pb. Because it contains biomolecules such as carbohydrates, lipids, amino acids, peptides, phenols, flavonoids, terpenes, and alkaloids, the *C. argentea* plant has hepatoprotective, antioxidant, anticancer, and antidiabetic effects (Malomo et al., 2011; Hamzah et al., 2018).

**TABLE 1:** Classification of *C. argentea* plant.

Kingdom	: Plantae (Angiosperms)
Super division	: Spermatophyte
Division	: Eudicots/Magnoliophyta
Class	: Core eudicots/Magnoliopsida
Order	: Caryophyllales
Family	: Amaranthaceae (flowering plants)
Subfamily	: Amaranthoideae and Gomphrenoideae
Genus	: Celosia (184 scientific plants, 51 species are accepted) and Lithophila
Species	: *C. argentea*
Binomial name	: *Celosia argentea* L., Amaranthus Cristatus
Synonyms	: *Celosia cristata*, *Semen celosiae*

Several ailments, including jaundice, inflammation, wound healing, eye diseases, and glandular swellings, had long been treated with *C*. *argentea* in traditional medicine. Numerous studies on *C. argentea* anticancer and antibacterial mechanism have shown evidence that it causes cells to commit suicide through apoptosis (Rub et al., 2015; Rub et al., 2016; Santana et al., 2021). Moreover, *C. argentea* is a powerful scavenging activity of free radicals. Therefore, this manuscript synthesized biogenic ZnO NPs using *C. argentea* extract. The physicochemical properties, such as size morphology, functional groups, and optical band gap, were determined. DPPH radical scavenging activity of *C. argentea* extract, chemically and biogenic ZnO NPs was assessed using DPPH assay and compared with ascorbic acid as standard. Agar disc diffusion was used to determine the zone of inhibition of biogenic ZnO NPs against *S. aureus*, *Bacillus subtilis*, *E. coli*, and *Salmonella typhimurium* bacterial strains. The cytotoxic effect of biogenic ZnO NPs against normal and cancer cell were determined using MTT assay. The cell cycle was analyzed using flow cytometry to investigate the impact of biogenic ZnO NPs on the apoptotic rate of HepG2 cells.

## 2 Materials and methods

### 2.1 Extraction from the leaves of *C. argentea*

The leaves of *C*. *argentea* were obtained from the South Valley University farm and rinsed with deionized water. The washed and dried leaves were cut into little pieces. 20 g of *C*. *argentea* were combined with 200 mL of deionized water and heated for 30 min at 80°C. After cooling to ambient temperature, the mixture was filtered, collected, and stored in a refrigerator at 4°C for future experiments.

### 2.2 Synthesis of ZnO NPs

**FIGURE 1:**
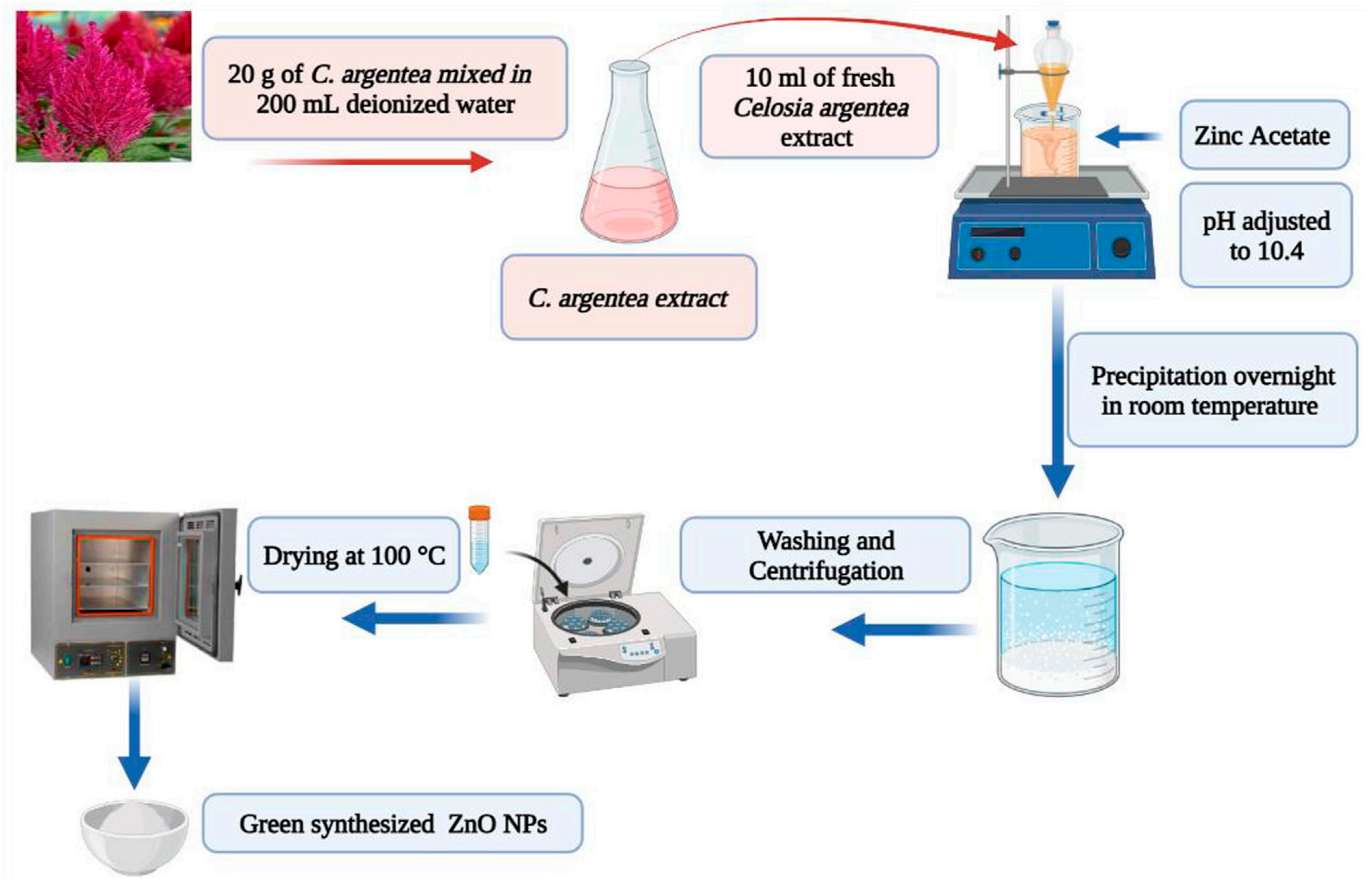
Schematic illustration of biogenic synthesis of ZnO NPs from *C*. *argentea* plant extract.

For comparison, two samples of ZnO NPs were synthesized under the same conditions: one with Sodium hydroxide as a reducing agent and the other with *C. argentea* extract. Two solutions were prepared following the coprecipitation approach. The precursor and reduction solutions in a typical synthesis, 5 g of zinc acetate [Zn(CH_3_CO_2_)_2_], are dissolved in 100 ml of deionized water and stirred (1500 RPM) for 30 min. The reduction solution was 0.1 M of sodium hydroxide in the case of chemical ZnO NPs and 10 ml of fresh *C. argentea* extract in the case of biogenic ZnO NPs. The reducing solution was added to the precursor and stirred for 1 h (1500 RPM). Sodium hydroxide and hydrochloric acid were used to adjust the pH of the mixture to 10.4, and the mixture was left under stirring for another hour. After precipitation overnight at room temperature, NPs were collected by centrifugation (5000 RPM, 5 min, at room temperature), washed ten times with distilled water and one more time with ethanol, and dried in an oven at 100°C for 6 h (Figure 1).

### 2.3 Characterization ZnO NPs

X-ray diffraction analysis was conducted using the X'Pert PRO-PAN instrument with a copper target (Cu-K = 1.54056 A) operating at 40 kV and 30 mA. Additionally, the structural features of biogenic ZnO nanoparticles were investigated using a transmission electron microscope (JEOL, JEM 2100, Japan). Optical properties were measured using infrared spectroscopy (FTIR Model 6100, Jasco, Japan) and UV-visible spectrophotometry (SPECORD 200 PLUS, Analytik Jena, Germany).

### 2.4 Free radical scavenging activity (DPPH assay)

Using a standardized (DPPH) assay, the free radical scavenging activity of biogenic ZnO NPs, *C. argentea extract*, and ascorbic acid was determined ^20,21^. The stock solution of three samples (green ZnO NPs, *C. argentea* extract, and ascorbic acid) was generated first by dissolving 4 g in 4 ml methanol, followed by eight serial dilutions (1,000, 500, 250, 125, 62.5, 31.25, 15.625, and 7.8125 mg/ml). Second, 3 ml of DPPH was added to each test tube and incubated in the dark for 30 min at room temperature. Finally, at 517 nm, the absorbance (A) of the control and samples were measured spectrophotometrically.

The assessment of scavenging ability against DPPH free radicals was conducted for each sample and expressed as a percentage concerning a reference standard from Reference. ^20^:

$$\text{Free radical scavenging activity}\left(\%\right)=\left(\frac{{\text{Abs}}_{\text{control}}-{\text{Abs}}_{\text{sample}}}{{\text{Abs}}_{\text{control}}}\right)\times 100$$ (1)

The IC_50_ was plotted as a function of concentration from a graph of free radical scavenging activity.

### 2.5 Antibacterial activity of biogenic ZnO NPs

The agar disc diffusion method described by Mounyr Balouiri et al. was used to test the antibacterial activity against different bacterial strains: gram-positive [*Staphylococcus aureus* (ATCC 29213), *Bacillus subtilis* (ATCC 6633)] and gram-negative [*E. coli* (ATCC 25922) and *Salmonella typhimurium* (ATCC14028)]. Four samples were prepared in normal saline (0.9% NaCl). Biogenic ZnO NPs samples were prepared by sonication to get a dispersed NPs solution and diluted into three concentrations (25, 50, and 100 μg/ml). For comparison, a chemical ZnO NPs sample was used with the high concentration (100 μg/ml)*, C. argentea* extract with concentration (20 μg/ml), and Gentamycin with concentration (20 μg/ml) as a positive control. Bacterial strains were cluttered in (Luria–Bertani) LB broth Overnight to obtain tested bacterial suspensions. Sterile Whatman filter paper discs were placed on the surface of the agar media, and then a proper volume containing the tested concentration of each sample was added. After incubating the plates for 24 h at 37°C, the diameters of the inhibitory zones were measured in millimeters. Each experiment was carried out three times to ensure accuracy.

### 2.6 Anticancer activity

#### 2.6.1 Cell viability measurement

Liver hepatocellular cells (HepG2) and Human umbilical vein endothelial cells (HUVEC) cell lines were obtained from the Egyptian holding company for biological products and vaccines (Vacsera), Giza, Egypt. Cell viability was screened against chemical ZnO NPs, biogenic ZnO NPs, *C. argentea* plant extract and doxorubicin at different concentrations using a standardized MTT assay (Kamal et al., 2022). Briefly, HepG2 (10,000 cells/well in a 96-well plate) was grown in complete DMEM culture media containing 10% heat-inactivated fetal bovine serum (FBS) and 1% penicillin-streptomycin and incubated for 24 h at 37°C, 5% CO_2_. The next day, the culture medium was cleaned with phosphate-buffered saline (PBS) to remove the detached cells. The culture media containing biogenic ZnO NPs (500, 250, 125, 62.5, 31.25,15.8, 7.8, 3.91.9,1.5, and 1 μg/ml) were added to cells and incubated for 24 h at 37°C, 5% CO_2_. At the end of the exposure time, cells were washed with PBS, 80 µL of media without FBS, and 20 µL of the MTT reagent. The reaction time for MTT assay was 3 h, then 100 µL of MTT solvent Dimethyl sulfoxide (DMSO) was added into each well and left for 15 min in the dark. The Analytik Jena UV spectrophotometer records the absorbance at 590 nm. Cell viability% is given by (Al-Harbi et al., 2023):

$$Cell\;viability\%=\frac{A_{Control}-A_{Sample}}{A_{Control}}$$ (2)

#### 2.6.2 Cell cycle analysis

HepG2 Cells (control) and cells after treatment with IC_50_ concentration of biogenic ZnO NPs were seed in DMEM medium overnight, then fixed in 70% (v/v) ethanol in PBS at 4°C for 12 h and re-suspended in PBS containing 40 μg/ml propidium iodide (PI), 0.1% (v/v) Triton X-100 and 0.1 mg/ml RNase in a dark room according to Juan-García et al. (2013) protocol Sub-G1 and cell cycle analysis was performed after a 30-min incubation at 37°C using a flow cytometer with an argon ion laser (wavelength of 488 nm).

Note: This work’s experimental research and field studies comply with relevant institutional, national, and international guidelines and legislation.

### 2.7 Statistical analysis

The data were processed using the Statistical Package for the Social Sciences (SPSS 25.0) software, and the results were expressed as the mean standard deviation. The data were analyzed using (ANOVA), and the *p* < 0.05 value indicated a statistically significant difference.

## 3 Results and discussion

### 3.1 Characterization ZnO NPs

**FIGURE 2:**
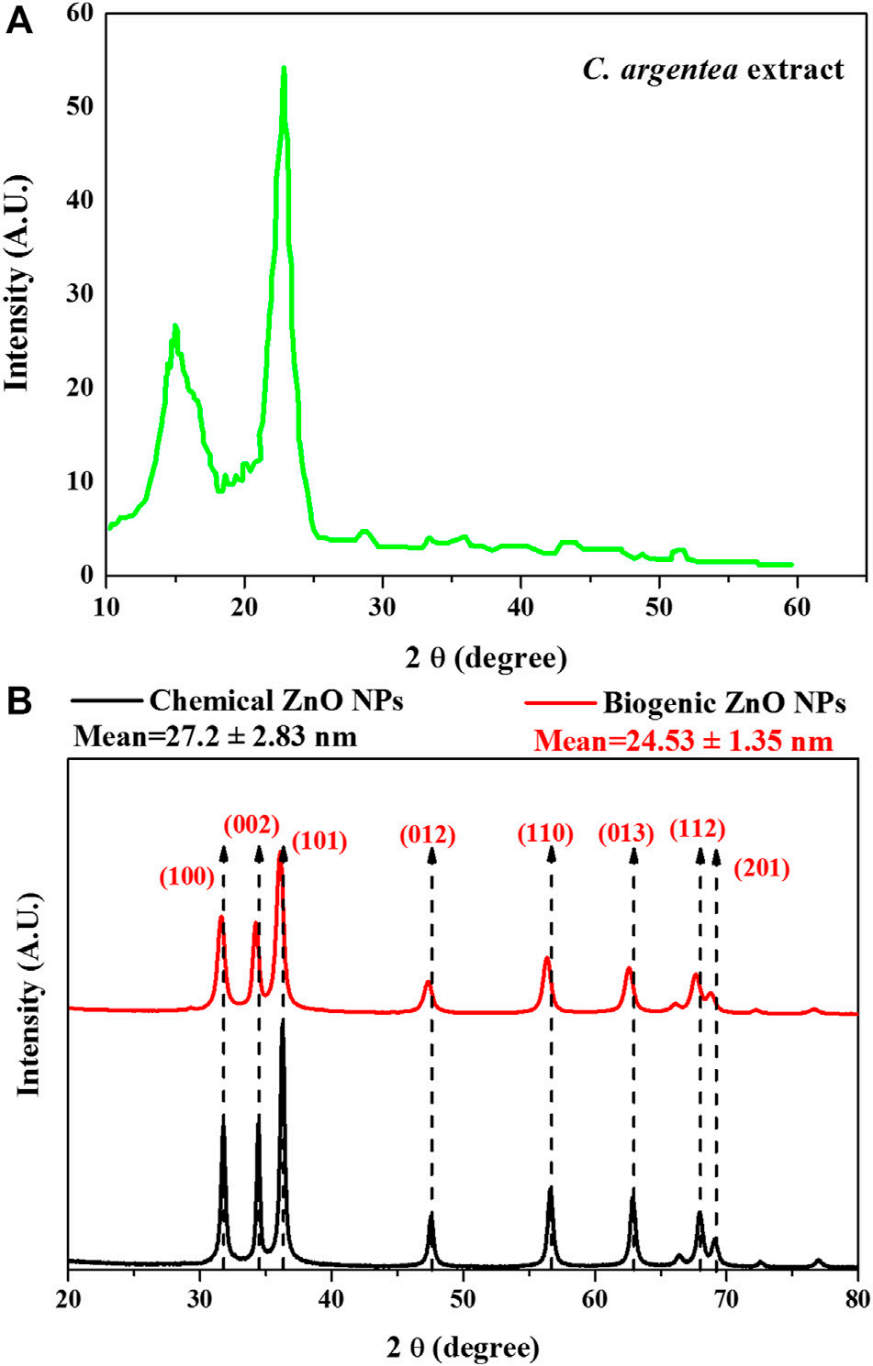
XRD pattern of *C. argentea* extract **(A)**, and chemical and biogenic ZnO NPs **(B)**.

Green synthesis of NPs is considered the most cost-effective, biocompatible, and flexible method for NPs synthesis. Biomolecules in the living organism have a high efficacy in reducing the metal ions to produce functionalized NPs with superior biocompatibility. To investigate the role of *C. argentea* extract on the formation of ZnO NPs, XRD analysis was performed for dried *C. argentea* extract, chemically and biogenic ZnO NPs. Figure 2A shows the XRD pattern of dried *C. argentea* extract. Mostly, the diffraction peaks at 2θ 14.97° and 22.88° belong to the fibrous cellulose (Manimaran et al., 2019; Ilaiya Perumal and Sarala, 2020). The successful formation of ZnO NPs was confirmed in both chemically and biogenic samples by the presence of fingerprint peaks in the XRD pattern, Figure 2B. The results in contact with JCPDS card No. 36-1451 for ZnO NPs (Wahab et al., 2014) and with the reported results of green ZnO NPs synthesized from different plants (Manokari and Shekhawat, 2017; Yusof et al., 2020; Sadhasivam et al., 2021). No additional peaks were observed for biomolecules from plant extract, indicating the absence of impurities and reflecting the prepared sample’s purity. Also, the sharp and narrow peak at 2θ ∼ 36° indicates the crystalline nature of the synthesized green ZnO NPs.

For chemical ZnO NPs, the diffraction peaks at 2θ (31.79, 34.43, 36.25, 47.54, 56.58, 62.84, 67.93 and 69.08) corresponding to the plane (100, 002, 101, 012, 110, 013, 112 and 201) respectively. A small shift in the peak position and decrease in the peak intensity was observed in the XRD pattern of biogenic ZnO NPs to be (31.5, 32.23, 36, 47.32, 56.42, 62.65, 67,82 and 69) corresponding to the plane (100, 002, 101, 012, 110, 013, 112 and 201) respectively. This peak shift was accompanied by a decrease in the peak intensity, suggesting that green synthesis decreases the crystallinity of ZnO NPs.

In the case of ZnO wurtzite structure *a* = *b* = 3.249 A° and *c* = 5.206 A°. The lattice constant *a* was calculated for the plane (100) using the relation (Gatou et al., 2023):

$$a=\frac{\lambda }{\sqrt{3}\sin\theta }$$ (3)

Lattice constant *c* calculated from for the plane (002) using the relation (Gatou et al., 2023):

$$c=\frac{\lambda }{\sin\theta }$$ (4)

The unit cell volume for the hexagonal structure was calculated using the following expression (Gatou et al., 2023):

$$V=\frac{\sqrt{3}}{2}a^{2}c$$ (5)

The calculated unit cell parameters for chemical ZnO NPs were almost identical to the reported standard values (*a* = 3.247 A°, *c* = 5.203 A°, and unit cell volume V = 47.53 A°). While biogenic ZnO NPs showed some expansion in all unit cell directions (*a* = 3.275 A°, *c* = 5.548 A° and unit cell volume V = 51.55 A°), Table 2.

**TABLE 2:** Summary of crystal structure of ZnO NPs calculated from XRD and HR-TEM analysis.

Structural property		Biogenic ZnO NPs	Chemical ZnO NPs
Lattice parameters	*a*	3.275	3.247
	*c*	5.548	5.203
	v	51.55	47.532
XRD analysis by the Debye-Scherer equation	D_L_ (nm)	24.53	27.206
	ɛ (10^−3^)	2.7	0.88
	SSA (m^2^ g^−1^)	43.6	39.38
XRD analysis by the Williamson-Hall method	D_L_ (nm)	20.4	25.088
	ɛ (10^−3^)	2.8	0.908
	SSA (m^2^ g^−1^)	52.4	42.7
HR-TEM analysis	D_L_ (nm)	21.55	26.48
	SSA (m^2^ g^−1^)	49.6	40.5

XRD pattern analysis is a method that is frequently utilized to analyze the structure of materials, particularly nanomaterials. The Debye-Scherer and Williamson-Hall techniques are two of the more common methodologies used in the XRD investigation of metal oxide nanomaterials. The Debye-Scherer method is used to calculate crystal size (***D***_***L***_). The approach presupposes that X-rays enter the material and interact with all its crystallites. Using the Debye-Scherer equation, the main diffraction pattern (101) is then utilized to calculate the crystal size and microstrain:

$$D_{L}=k\lambda /\beta \cos\theta$$ (5a)

ɛ=βcosθ/4 (6)

Where k is a constant, *λ* wavelength, *β* diffraction peak, and *θ* is the Bragg angle.

**FIGURE 3:**
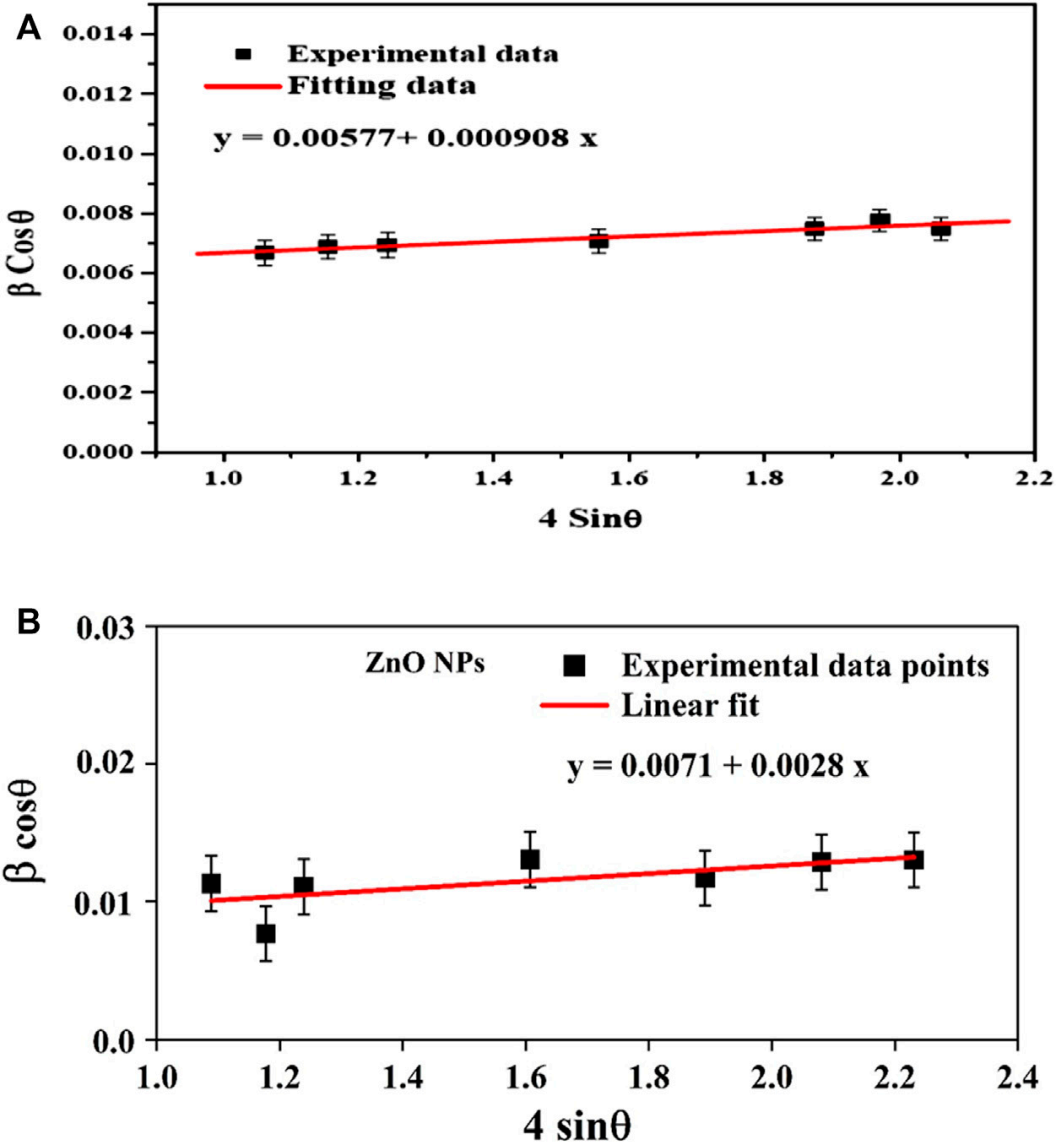
The W-H plot for chemical ZnO NPs **(A)** and biogenic ZnO NPs **(B)**.

In contrast, the Williamson-Hall method considers the impact of crystal size and lattice strain on the diffraction pattern. The approach posits that the X-rays interact with a finite number of crystallites in the sample and that the broadening of the diffraction peaks is caused by both the crystallites’ finite size and the presence of lattice strain. According to this model, the average crystallite size (D_L_) and average micro-strain (ε) were found by taking the reciprocal of the intercept of (β cos θ) versus (4 sin θ) (Figures 3A, B). The primary distinction between the two methodologies lies in their underlying assumptions. The Debye-Scherer technique operates under the premise that the observed diffraction pattern is exclusively attributable to the finite dimensions of the crystallites. Conversely, the Williamson-Hall method considers the impact of crystal size and the influence of lattice strain on the resulting diffraction pattern.

**FIGURE 4:**
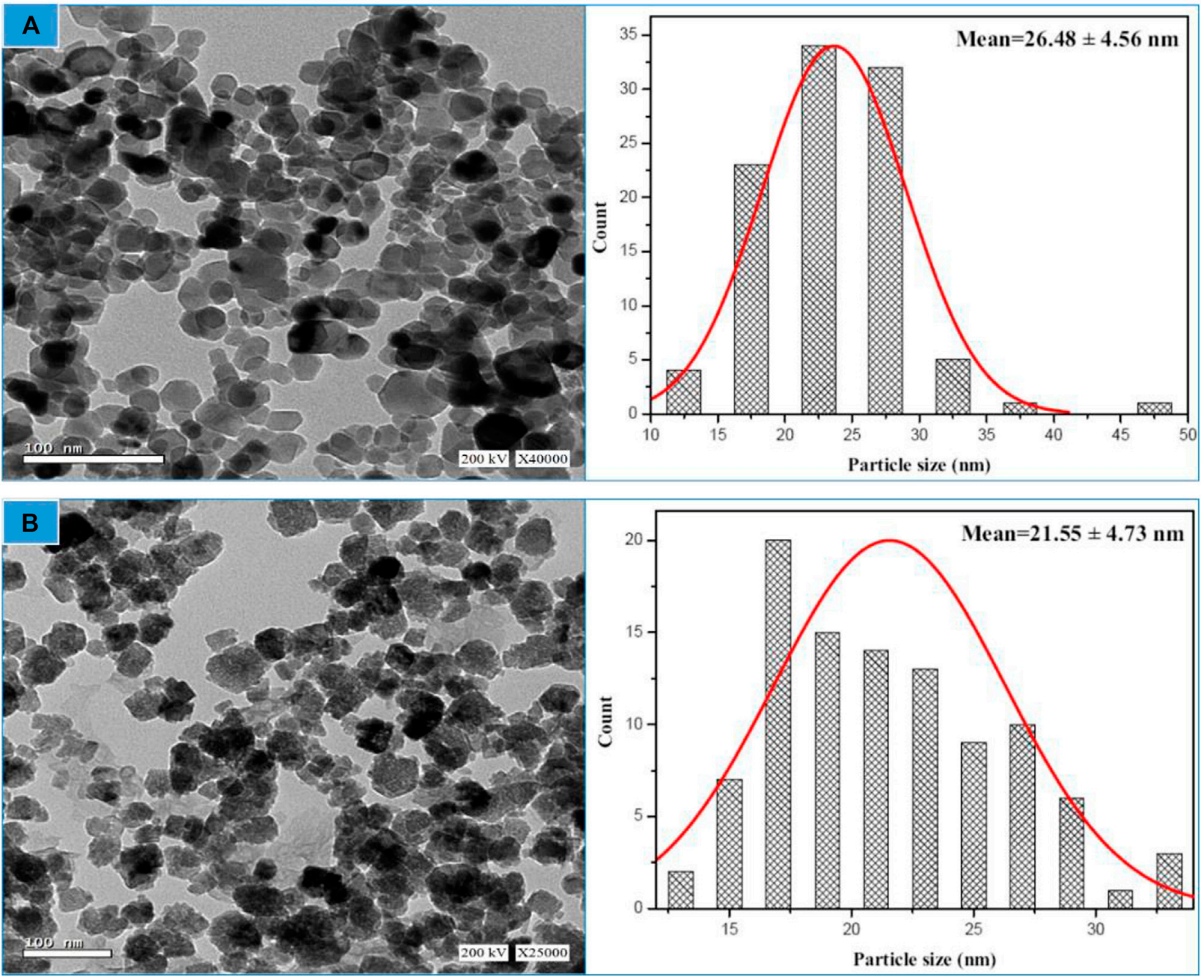
HR-TEM image linked with particle size distribution for chemical ZnO NPs **(A)** and biogenic ZnO NPs as determined from HRTEM image (*n* = 100) **(B)**.

The synthesized biogenic ZnO NPs were imaged by a high-resolution transmission microscope (HRTEM), Figures 4A, B. Chemical ZnO NPs showed a non-uniform spherical shape. While an accumulation of the formed semispherical particles of biogenic ZnO NPs was observed. It is reported that using biomolecules as reducing agents increases particle aggregation due to the electrostatic interaction between metal ions and biomolecules. Similar results were obtained for the biosynthesis of green ZnO NPs from different plant extracts (Manokari and Shekhawat, 2017; Yusof et al., 2020; Sadhasivam et al., 2021). ImageJ software was used to estimate the particle size from HETEM images. The number of counted particles was 100 per image. The average particle size was 26.48 ± 4.56 and 21.55 ± 4.73 nm for chemical and biogenic ZnO NPs, respectively.

The following expression can be used to calculate the specific surface area (SSA), which is defined as the total surface area per unit mass and is applicable for adsorption and reactions that take place on the surface of the material:

$$\text{SSA}=S_{f}\;/\beta .\;Ρ$$ (7)

S_f_ represents the form factor of ZnO NPs, *β* is the mean particle size, and ρ is the density of ZnO (5.61 g/cm^−3^).

The SSA for biogenic ZnO NPs is shown in Table 2 using the assumption that the particles are spherical (S_f_ = 6), where particle sizes were measured using different methodologies.

From the structural analysis, it can be concluded that the biogenic synthesis of ZnO NPs from *C. argentea* extract didn’t change the structure of ZnO NPs. However, the produced NPs possess smaller size, lower microstrain, and higher specific surface area than chemically synthesized ZnO NPs.

The antioxidant, antibacterial, and anticancer capabilities of ZnO NPs can be improved by increasing their specific surface area and decreasing their particle size value. The larger number of reactive sites on the surface of ZnO NPs enhances their ability to scavenge free radicals and their antioxidant activity (Ramesh et al., 2022). The increased surface area of the NPs prevents oxidative damage to cells and tissues by efficiently adsorbing free radicals. Accordingly, this promises to treat various medical issues, including cardiovascular illness, neurological disease, and cancer (Hamed et al., 2023). ZnO nanoparticles (NPs) have a greater surface area relative to their volume, increasing the interaction between the NPs and bacterial cells and leading to greater antibacterial activity. This improved connection can potentially increase the NPs’ antibacterial activity by facilitating their penetration of the bacterial cell wall and disrupting cellular processes. As a result, such materials are used in the development of antibacterial coatings for use on medical equipment and surfaces and in the management of bacterial infections.

To generate reactive oxygen species (ROS) and cause cellular damage in cancer cells, ZnO NPs with a high specific surface area induce apoptosis (programmed cell death). The enhanced surface area of the nanoparticles facilitates more effective production of reactive oxygen species (ROS), enhancing their anticancer efficacy (Snezhkina et al., 2019). The large surface area can penetrate the tumor microenvironment and home in cancer cells more easily while avoiding collateral damage to healthy tissue. The process may help in the development of more precise tumor-targeting cancer medicines.

**FIGURE 5:**
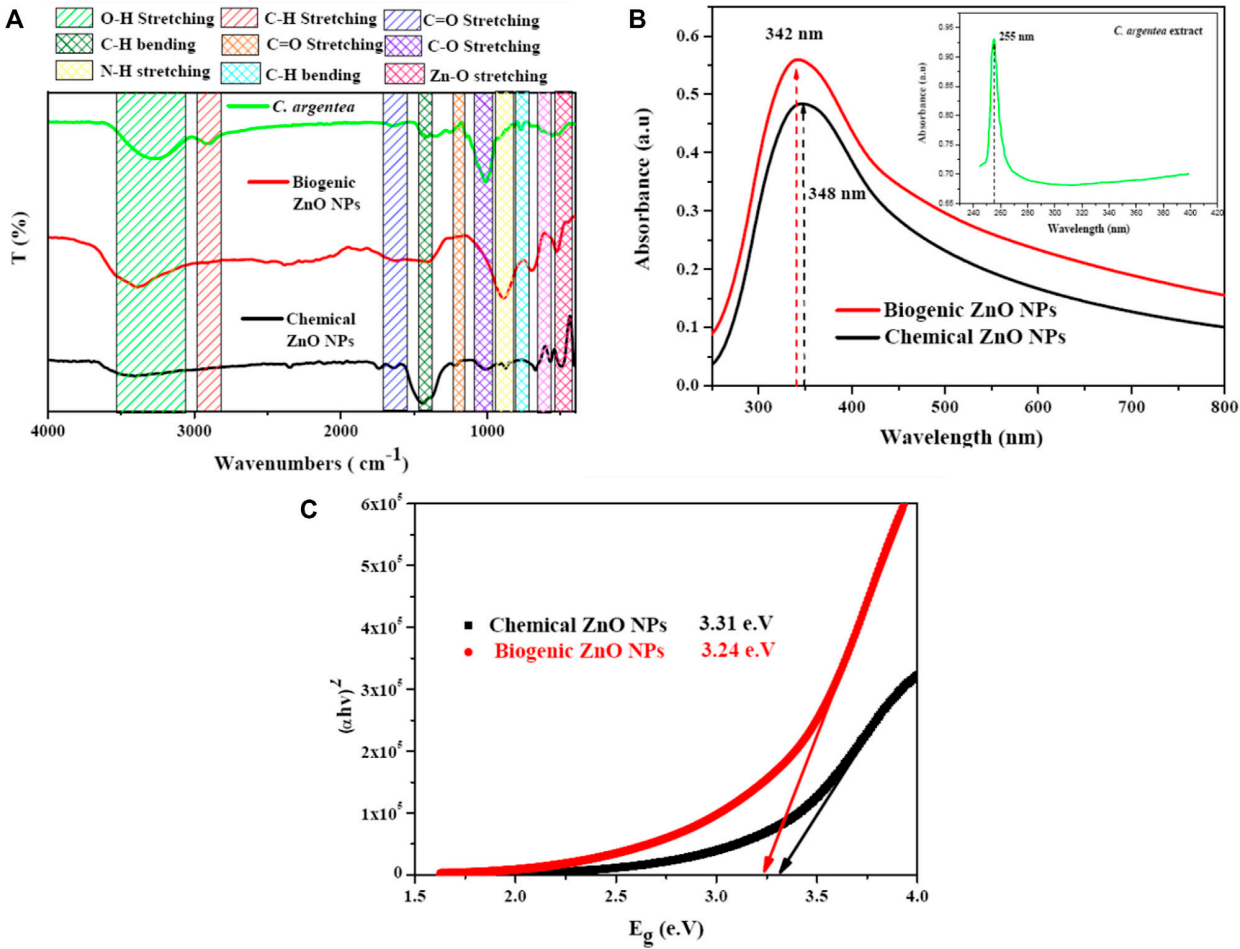
Optical spectroscopy of *C. argentea*, chemical and biogenic ZnO NPs; FTIR spectrum **(A)** UV-vis spectrum **(B)**, and band gap energy calculation **(C)**.

Fourier transformation Infrared spectroscopy (FTIR) was employed to investigate the possibility of Zn ions reduction and the formation of ZnO NPs. Several absorption bands were observed due to the presence of ZnO NPs and the organic molecules of *C. argentea* Figure 5A. Vibrational bands were observed at 452 and 526 cm^−1^, corresponding to Zn-O stretching vibration. The absorption bands appearing at 698, 1,410, and 2,384 cm^−1^ are due to C-H bending vibrations, while the band at 890 cm^-1^ can be attributed to N-H stretching. C=C stretching vibration appeared at 1,620 cm^−1^, while C=O appeared at 2,508 cm^−1^.

Finally, the broad band observed was approximately between 3,000 and 3,500 cm^−1^ corresponding to the OH vibration of the hydroxyl group. The formation of ZnO NPs was confirmed by the presence of Zn-O vibrational bands at 452 and 526 cm^−1^ in the FTIR spectra, which are the fingerprints of inorganic NPs (Yusof et al., 2020; Sadhasivam et al., 2021). Some functional groups were detected due to the interaction of Zn^+2^ ions with the biomolecules of *C. argentea* leaves, suggesting the successful reduction of ZnO and the formation of ZnO NPs (Parvathy et al.). Figure 5B shows the absorption spectrum of *C. argentea*, chemical, and biogenic ZnO NPs with an absorption peak at 255, 248, and 342 nm, respectively (Press et al., 2017). The optical band gap was calculated using the Tauc equation as the following (Gatou et al., 2023):

$$\alpha h\upsilon =A{\left(h\upsilon -E_{g}\right)}^{n}$$ (8)

where *α* is the absorption coefficient, *h* is the blank constant, A is the proportional constant, and E_g_ is the band gap energy. Herein, the value of constant n depends on the type of the transition process; for direct transition, n = 2, while n 1/2 for indirect optical transitions. For ZnO NPs, the possible transition is indirect, so that n will be considered = 1/2 (Rahman et al., 2020). Plotting the relation between (αhυ)^2^ and the incident photon energy Eg (hυ), the band gap energy can be calculated by extrapolating the linear region on the energy axis, Figure 5C. The calculated band gap energy was 3.31 and 3.24 e.V for chemical and biogenic ZnO NPs, respectively. These values are smaller than the reported value (3.37 e.V). The decrease can be explained as the biosynthesis with plant extract modifying the surface of ZnO NPs, resulting in increased electron densities and lower energy bands (Alharbi et al., 2023).

### 3.2 Free radical scavenging activity

The antioxidant activity of biogenic ZnO NPs is attributed to their ability to donate hydrogen. The formation of electron-hole pairs on the surface of ZnO NPs has great potential for reducing H_2_O molecules, which can work as scavengers of DPPH molecules (Bisht and Rayamajhi, 2016). However, the biogenic synthesis of ZnO NPs can contribute to modulating the scavenging ability due to phytochemicals such as phenolics and polyphenolic compounds in the plant extract (Madan et al., 2016). These phytochemicals have antioxidant abilities, which contribute to the inhibition of free radical scavenging.

**FIGURE 6:**
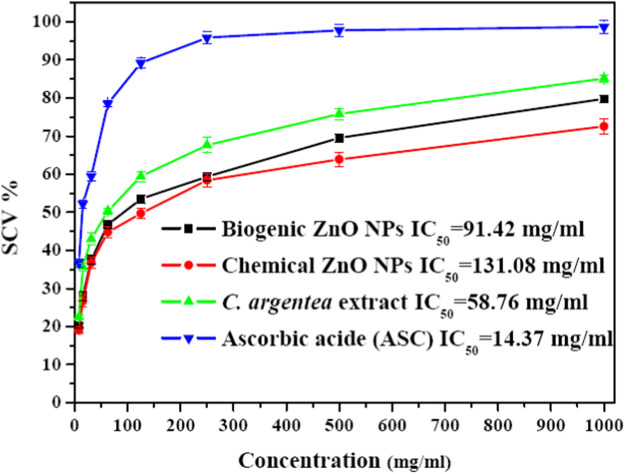
Savaging activity of chemical and biogenic ZnO NPs from *C. argentea*, *C. argentea* aqueous extract of *C. argentea*, and Ascorbic acid as evaluated from DPPH assay.

Figure 6 shows the DPPH radical scavenging activity of biogenic ZnO NPs compared with chemical ZnO NPs *C. argentea* and ascorbic acid (ASC) as standards. These results reflect a dose-dependent behavior in the scavenging activity of biogenic ZnO NPs with Ic_50_ = 91.24 μg/ml compared with Ic_50_ = 131.08, 58.76, and 14.37 μg/ml for chemical ZnO NPs, *C. argentea* and ascorbic acid, respectively. Similar results were reported for green ZnO NPs synthesized using the DPPH assay in Table 3. The Ic_50_ of the aqueous extract *of C. argentea* was 30 μg/ml, which was attributed to its phenolic content as reported by Rub et al. (2013). Hence, the biosynthesis of ZnO NPs using *C. argentea* extract enriched its surface with biomolecules and improved its scavenging activity.

**TABLE 3:** Maximum savaging activity of biosynthesized ZnO NPs from different plants using DPPH assay.

Plant name	Maximum activity	References
*Knoxia sumatrensis*	95.8%	Loganathan et al. (2021)
*Azadirachta indica*	92%	Madan et al. (2016)
*Rosa canina*	>90%	Jafarirad et al. (2016)
*L. pruinosum*	86.5%	Naiel et al. (2022)
*Eucalyptus globulus*	82%	Siripireddy and Mandal (2017)
*Trianthema portulacastrum*	75%	Khan et al. (2019b)
*Costus igneus*	75%	Vinotha et al. (2019)
*Tecoma castanifolia*	67%	Sharmila et al. (2019)
*Andrographis paniculata*	61.32%	Rajakumar et al. (2018)
*Ceropegia candelabrum*	55.43%	Murali et al. (2017)

### 3.3 Antibacterial activity

**FIGURE 7:**
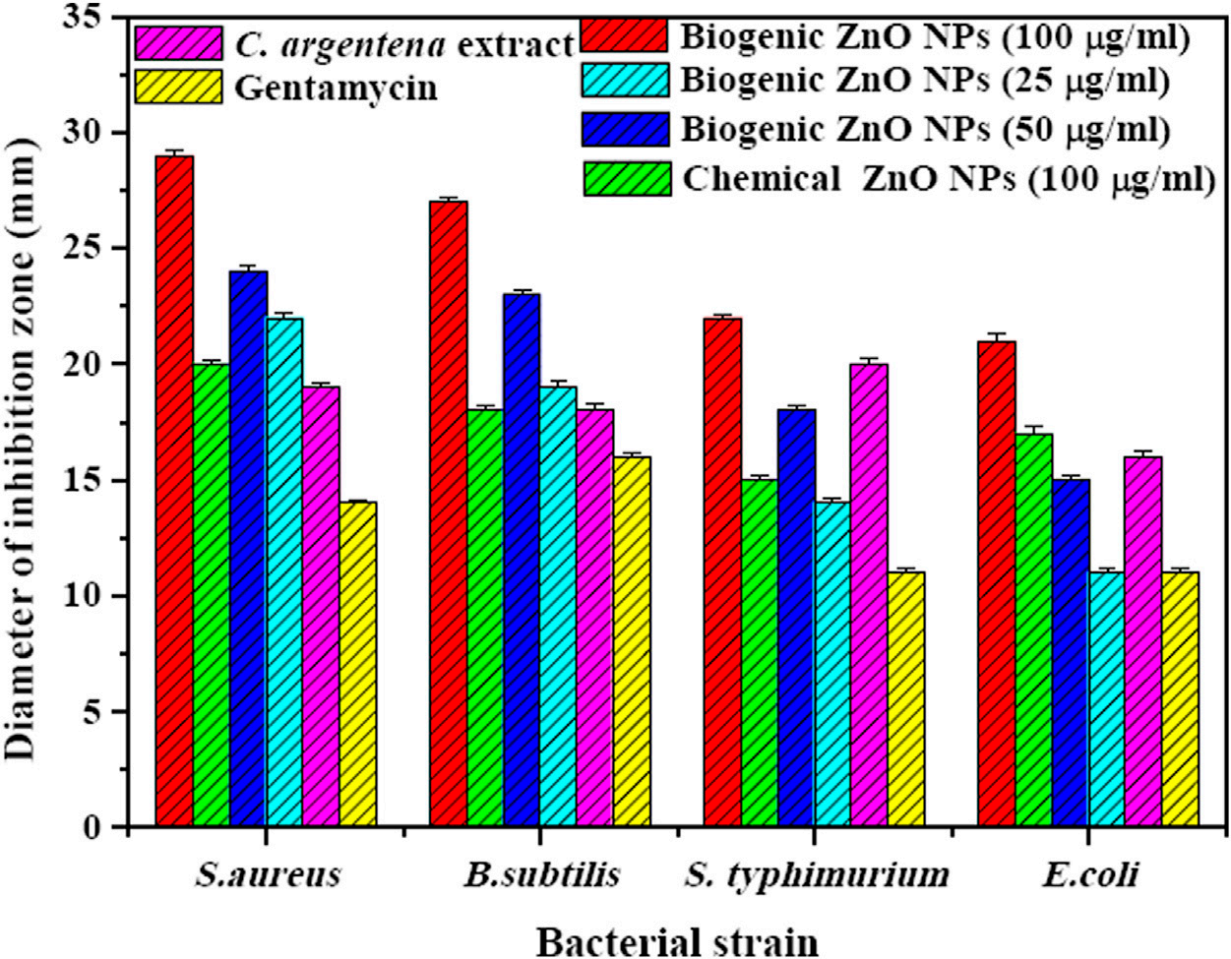
Diameter of inhibition zone (mm) of biogenic ZnO NPs with different concentrations (100, 50, and 25 μg/ml), chemical ZnO Nps (100 μg/ml), *C. argentea* extract (20 μg/ml), and Gentamycin (20 μg/ml) against gram-positive bacteria (*S. aureus* and *B. subtilis*) and gram-negative bacteria (*E. coli* and *Salmonella typhimurium*).

In this study, the antibacterial activity of biogenic ZnO NPs from *C. argentea* extract was evaluated against four types of bacterial strains: two gram-positive [*Staphylococcus aureus* (ATCC 29213), *Bacillus subtilis* (ATCC 6633)] and two gram-negative [*E. coli* (ATCC 25922) and *Salmonella typhimurium* (ATCC14028)]. The diameter of the inhibition zone of green ZnO NPs, chemical ZnO NPs, *C. argentea* extract, and Gentamycin as a positive control was determined using an agar disc diffusion method and presented in Figure 7. The highest concentration (100 μg/ml) was chosen for chemical ZnO NPs in order to compare its antibacterial activity. Biogenic ZnO NPs showed significant antibacterial activity against the four bacterial strains compared with the positive control and chemical ZnO NPs. Similar activity had been reported for green synthesized ZnO NPs from different plants (Table 4). Moreover, the inhibition zone of *C. argentea* extract against the four bacterial strains was significantly larger than that of Gentamycin, which was due to the presence of bioactive compounds such as alkaloids, flavonoids, phenols, terpenoids, starch, and cellulose (Malomo et al., 2011; Santana et al., 2021).

**TABLE 4:** Antibacterial activity of green ZnO NPs from different plants against different bacterial strains.

Plant source	Bacterial strain	Antibacterial activity (μg/mL)	Inhibition zone (mm)	References
*Cassia fistula*	*Escherichia coli*	20	4.67	Suresh et al. (2015)
*Plectranthus amboinicus*	*Staphylococcus aureus*	8-10	11-13	Vijayakumar et al. (2015)
*Vitex negunda*	*Staphylococcus aureus*	15	19	Ambika and Sundrarajan (2015)
	*Escherichia coli*	17	16	
*Ceropegia candelabrum*	*Staphylococcus aureus*	0.25	20.13	Murali et al. (2017)
	*Bacillus subtilis*		22.40	
	*Escherichia coli*		20.40	
	*Salmonella typhi*		19.30	
*Celosia argentea*	*Escherichia coli*	3.1	12	Vaishnav et al. (2017)
	*Salmonella*		17	
	*Acetobacter*		13	
*Couroupita Guianensis*	*Bacillus cereus*	0.05-0.25	*16.5*	Sathishkumar et al. (2017)
	*Klebsiella pneumonia*		*18*	
	*Escherichia coli*		*19*	
	*Mycobacterium luteus*		*15*	
	*Staphylococcusparatyphi*		*17.3*	
	*Vibrio cholerae*		*18.2*	
*Caulerpa peltata, Hypnea valencia, Sargassum and myriocystum*	*Staphylococcus aureus*	0.1	<5	Nagarajan and Arumugam Kuppusamy (2013)
	*Streptococcus mutans*		>5	
	*Vibrio cholerae*		>5	
	*Neisseria gonorrhoeae*		<5	
	*Klebsiella pneumonia*		>10	

Regarding the bacterial strain type, biogenic ZnO NPs showed a higher bactericidal effect in gram-positive strains (*S. aureus* and *B. subtilis*) than in gram-negative strains (*E. coli* and *Salmonella typhimurium*). The explanation of this result was investigated by Vijayakumar et al. and attributed to the differences in the structure and components of gram-positive bacteria, such as the peptidoglycan layer, which facilitates the attachment and internalization of ZnO NPs to the cell wall (Sharma and Kaushal, 2022). The same behavior was observed in the case of chemical ZnO NPs, except that the diameter of the inhibition zone was smaller.

Additionally, the inhibition zone of biogenic ZnO NPs increased dramatically with concentration increments from 25 to 100 μg/ml in the four bacterial strains. This behavior is due to the increased number of ZnO NPs diffused in the agar medium, which is in contact with the reported results (Singh et al., 2018; Sadhasivam et al., 2021; Publishers, 2023).

**FIGURE 8:**
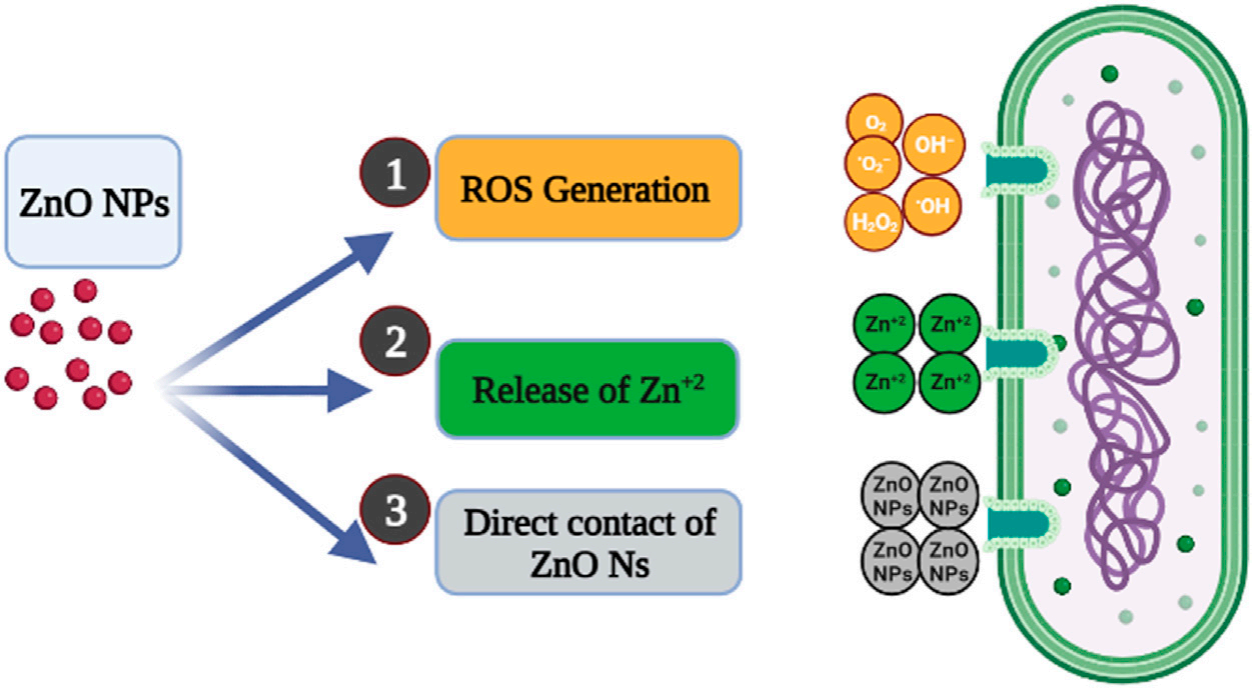
The possible antibacterial mechanism of ZnO NPs.

Several mechanisms were reported for the antibacterial activity of green ZnO NPs; the main three possible mechanisms were illustrated in Figure 8 (Ambika and Sundrarajan, 2015; Mendes et al., 2022; González et al., 2021). The proposed mechanisms include:

• The release of Zn^+2^ is due to the interaction of ZnO NPs with the bacterial cell. The negatively charged bacterial membrane attracted the released Zn^+2^ via Van der Waals forces, leading to defragmentation of the cell membrane, inhibition of cell growth, and finally cell death.
• The generation of reactive oxygen species (ROS), including hydrogen peroxide, radical hydroxyl, and peroxide, is from the surface of ZnO NPs. The ROS generated causes oxidative stress by damaging the cell membrane, DNA, and proteins, resulting in cell death.
• Direct contact with the bacterial cell membrane is another mechanism for the antibacterial action of ZnO NPs. According to Heinlaan et al. and Brayner et al., the contact of bacterial cells with ZnO NPs can cause a change in the contact area of the cell membrane, increasing the membrane permeability and leading to the subsequent internalization of ZnO NPs and cell damage.

The physiochemical properties such as size, shape, surface chemistry, and high surface-to-volume ratio are responsible for the antibacterial action of green ZnO NPs on different bacterial strains (Table 4).

### 3.4 Anticancer activity

One of the main advantages of ZnO NPs as an anticancer agent is their selectivity for cancer cells. The ability of NPs to kill cancer cells varies depending on their intrinsic properties, such as their ability to produce ROS. These ROS interact with the cellular components and initiate apoptosis. From a genetic point of view, apoptotic induction can occur through intrinsic or extrinsic pathways due to the activation of caspases. Although many genes can induce apoptosis, p53 is the master guardian of the cell, maintaining genomic stability through activated cell-cycle checkpoints, DNA repair, and apoptosis (Bisht and Rayamajhi, 2016; Tabrez et al., 2022). Cell cycle arrest is initiated by DNA damage and the p53 gene, enabling the possibility of either self-mediated programmed cell death or repair of the damage (Król et al., 2017; Levy et al., 2017).

The Bcl-2 level reduces resistance to apoptotic stimuli, resulting in apoptosis (Tang et al., 2017). Intrinsic pathways of apoptosis are the source of the mitochondria-integrated signals. The cell death resistance is motivated by the activation of NF-κB, which leads to downstream target gene expression (Albensi and Braun, 2019). The NF-κB activation in the mitochondria leads to cytochrome c release, thus triggering caspase cascades and programmed cell death.

#### 3.4.1 Cytotoxicity of ZnO NPs

**FIGURE 9:**
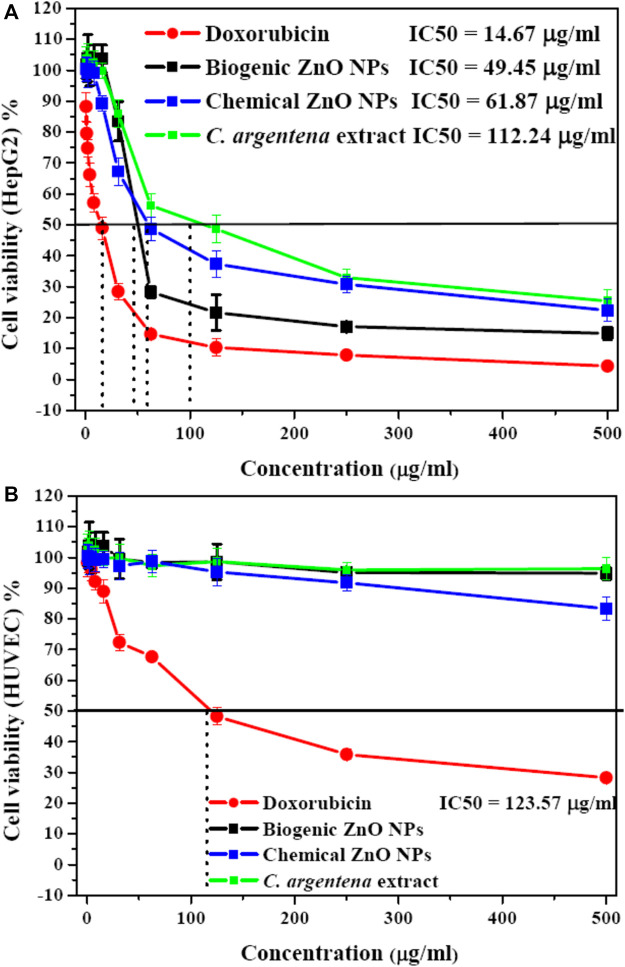
Cytotoxicity as screened with MTT assay of *C. argentena* extract, doxorubicin as a positive control, and chemical and biogenic ZnO NPs against HepG2 cells **(A)** and HUVEC cells **(B)**.

MTT assay was used to determine the cytotoxicity of biogenic ZnO NPs, chemical ZnO NPs, and *C. argentena* extract doxorubicin as positive control and with different concentrations (500, 250, 125, 62.5, 31.25,15.8, 7.8, 3.9, 1.9, 1, and 0.5 μg/ml) against HepG2 and HUVEC cells. The viability of HepG2 cells decreased in a concentration-dependent manner from (100%–14.9%, 88.27%–4.26%, and 103.73%–25.37%) with the increasing concentrations of biogenic ZnO NPs, doxorubicin and *C. argentena* extract, respectively from 0.5 to 500 μg/ml (Figure 9A). However, compared with doxorubicin, cell viability reduction was slightly small in the small concentrations from 0.5 to 15.8 μg/ml, while it increased in the higher concentrations from 15.8 to 500 μg/ml for biogenic ZnO NPs and *C. argentena* extract. The calculated IC_50_ was found to be 49.45, 14.67, and 112.24 μg/ml for biogenic ZnO NPs and doxorubicin and *C. argentena* extract, respectively. In normal cells (HUVEC), the only observed toxic effect belonged to doxorubicin with IC_50_ = 123.57 μg/ml and a small reduction in the high concentration of chemical ZnO NPs about 18% (Figure 9B). This finding reflects the powerful anticancer potential and higher selectivity of biogenic ZnO NPs against cancer cells. Additionally, these results supported the previous studies on the anticancer activity of biogenic ZnO NPs, and they revealed that *C. argentea* extract could be used as a reducing agent for ZnO NPs synthesis with high selectivity for cancer cells and improved anticancer activity (Raffa et al., 2011; Alrokayan et al., 2012; Bisht and Rayamajhi, 2016; Król et al., 2017).

#### 3.4.2 Cell cycle analysis

**FIGURE 10:**
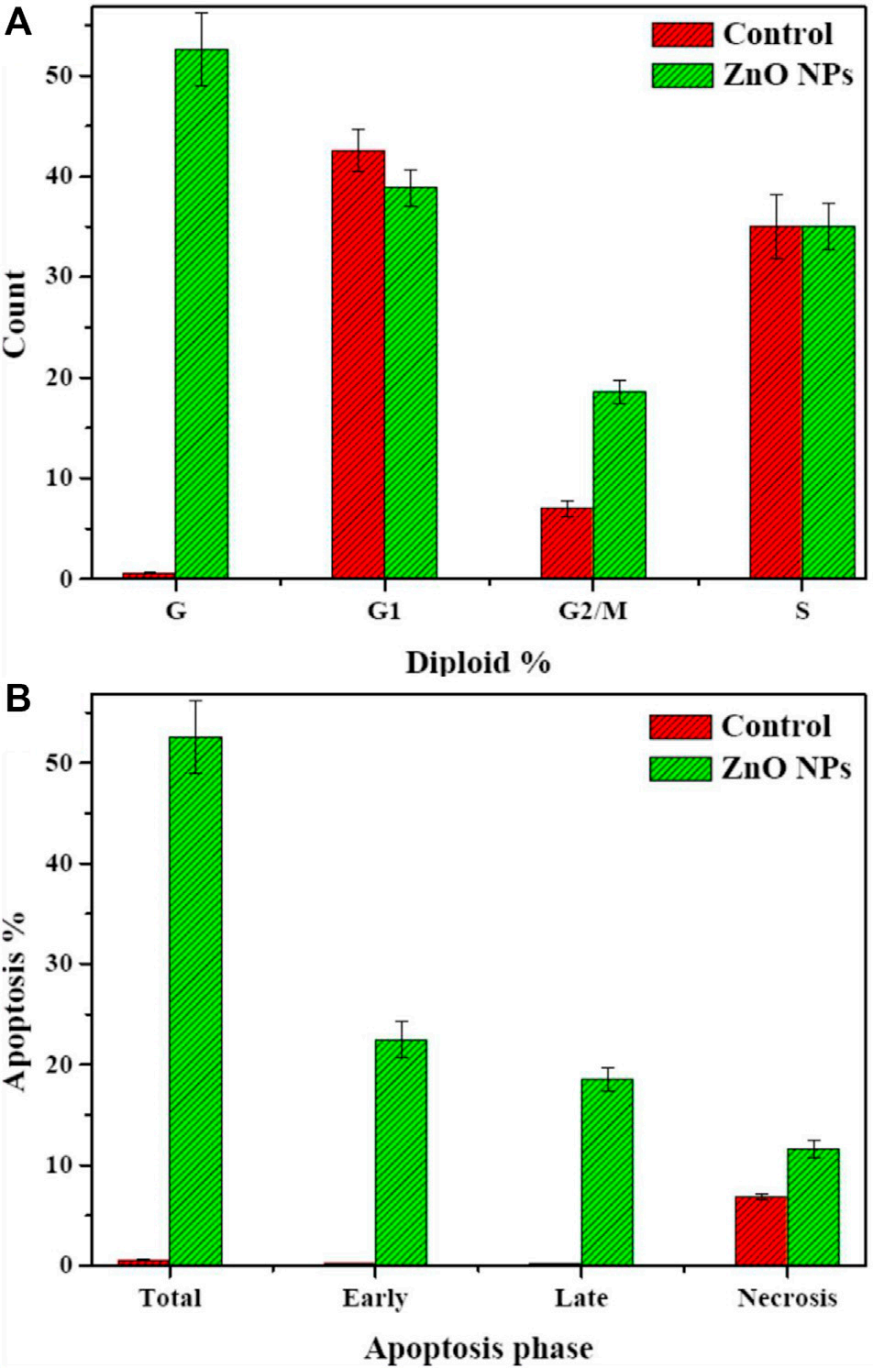
Flow cytometry. Cell cycle of HepG2 cells after treatment with biogenic ZnO NPs for 24 h. SubG1, S, and G2/M represented hepG2 cells in normal phases. In contrast, cells undergo apoptosis/necrosis represented in the G phase. **(A)** Analysis of necrosis and apoptosis percentage (Total, Early, and Late) of HepG2 cells before and after treatment with biogenic ZnO NPs **(B)**. *Statistically significant difference as compared with the controls (P, 0.05 for each. HepG2).

To determine the possible mechanism for the anticancer activity of biogenic ZnO NPs, flow cytometry was used to analyze the cell cycle of HepG2 cells. Apoptotic rate of treated cells with biogenic ZnO NPs was elevated for S phase 39.28%, G1/G0 was 52.68%, 8.04% for the G2/M phase and 52.6 for Pre-G1 compared with cell control values of 52.78%, 42.58%, 6.8%, 0.58%, respectively (Figure 10). Also, there was a significantly elevated (*p* < 0.05) necrotic cells % compared with the control value (11.61%/6.84%).

**FIGURE 11:**
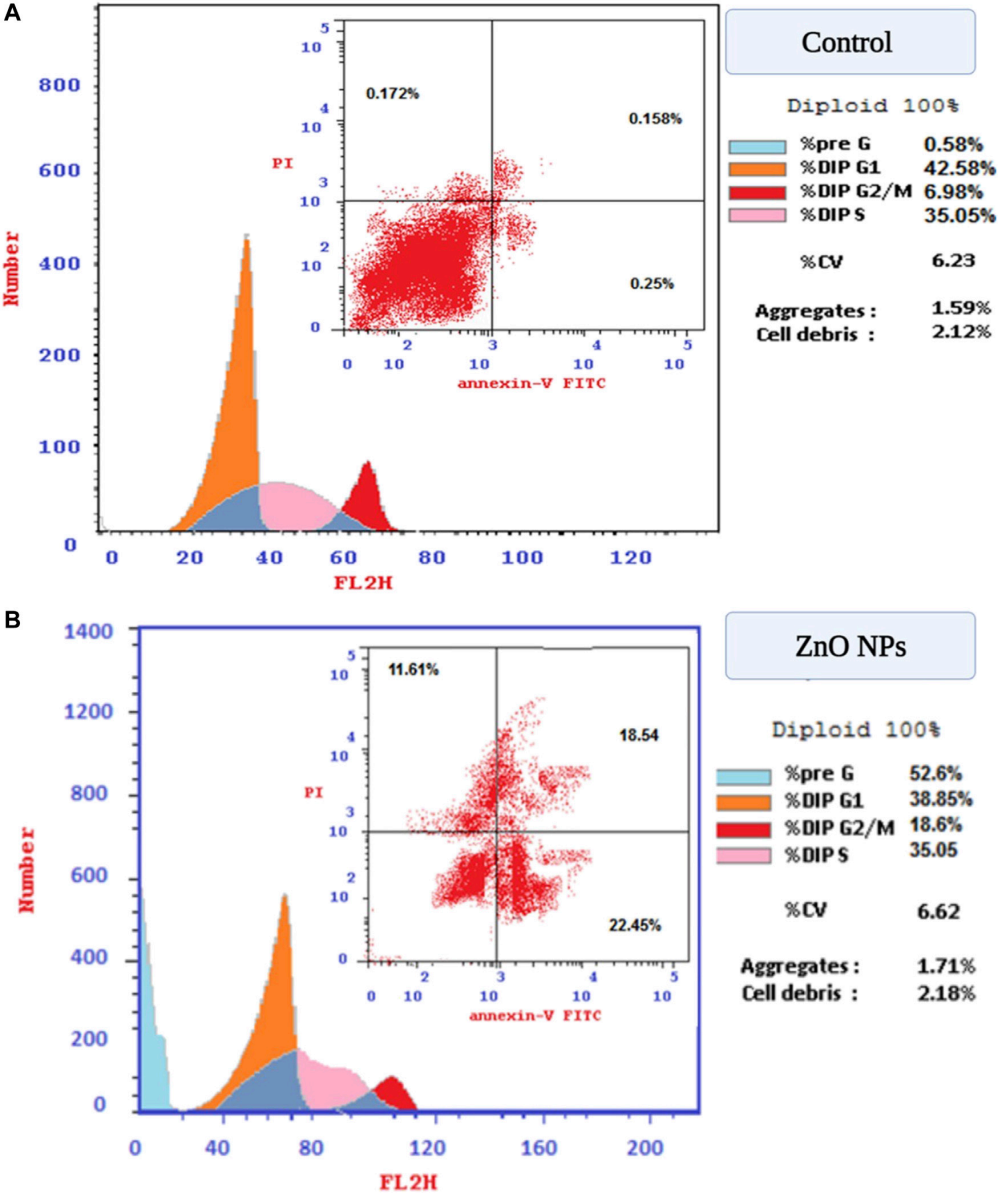
**(A)** Flow cytometry for evaluation of cell cycle profile using PI and annexin V. stains on HepG2 cells. **(B)** Post-treatment with green ZnO NPs on HepG2 cells.

An observed accrual of arrested cells in the G2/M phase with an increase in apoptotic cells appeared in the higher number of cells in the PreG1 phase (Figure 11). Moreover, the apoptotic cells showed a significant elevation in early and late apoptosis (22.45%, 18.54%) compared with control values (0.25%, 0.158%).

According to the literature, the cytotoxic effect of ZnO NPs can be attributed to two main mechanisms: necrosis and apoptosis. Exposure to ZnO NPs can induce the production of ROS or mediate protein activity disequilibrium. The oxidative stress generated can lead to necrosis or DNA damage. As a result of DNA damage, the mitochondrial apoptotic (p53 gene) pathway will be activated, causing cell death by apoptosis (Alrokayan et al., 2012; Wang et al., 2015; Press et al., 2017).

#### 3.4.3 RT-qPCR analysis

**FIGURE 12:**
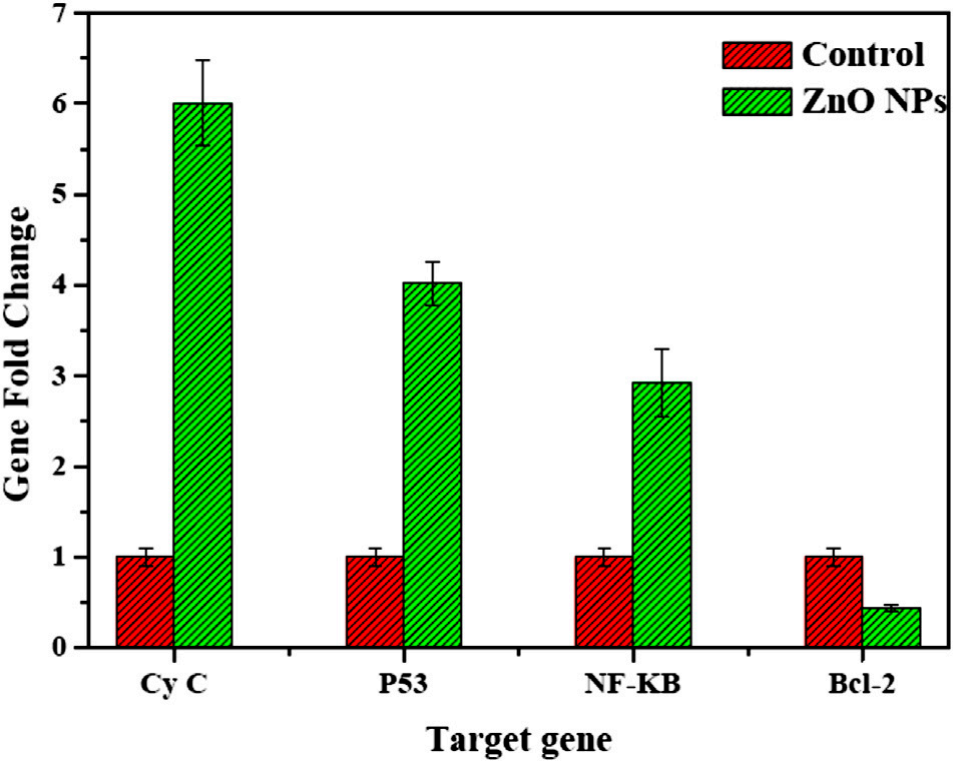
mRNA levels of proapoptotic and antiapoptotic genes HepG2 cells treated with biogenic ZnO NPs.

The mRNA expression levels significantly increased in Cy-c, p53, and NF-KB genes. In contrast, the expression of the antiapoptotic gene bcl-2 was significantly decreased compared with its values in untreated control HepG2 cells (Figure 12).

Our study exhibited the expressions of the proapoptotic NF-KB–Cy-c genes, the master guardian of the cell p53 gene, and the antiapoptotic Bcl2 gene, suggesting that biogenic ZnO NPs enter the cell by changing the mitochondrial membrane potential and activating the expression of the important tumor suppressor gene leading to an increase in its value (Wang et al., 2015; Moghaddam et al., 2017; Press et al., 2017).

Similar results were reported for apoptotic induction after treatment with biogenic ZnO NPs. Moghaddam et al. (2017) investigated the apoptotic induction of biogenic ZnO NPs, and they observed the downregulation of antiapoptotic genes of Bcl-2, AKT1, and JERK/2 and the upregulation of proapoptotic genes of p21, p53, JNK, and Bax in Pichia kudriavzevii GY1 yeast after treatment with biogenic ZnO NPs. Moreover, Alrokayan et al. (2012) reported the upregulation of p53 and bax genes and the downregulation of the antiapoptotic gene Bcl-2 in HepG2 cells after treatment with ZnO NPs. In another study, ZnO NPs synthesized from *E. prostrata* were found to promote autophagy, upregulate the expression of p53 and Caspase3, and trigger apoptosis (Chung et al., 2015). ZnO NPs synthesized from *Artemisia scoparia* extract upregulated proapoptotic genes while downregulating antiapoptotic genes (Shivyari et al., 2022).

According to the results, the anticancer activity of biogenic ZnO NPs is caused by the activation of apoptotic protease activator, which decomposes DNA polymerase and damages cell DNA, consistent with the reported results (Koopman et al., 1994; Jolla et al., 1995; Wahab et al., 2014).

## 4 Conclusion

Biogenic synthesis of NPs is a powerful technique to produce NPs with unique properties. The biomolecules of C. *argentea* extract assessed the reduction of Zn ions. The reduction process didn't affect the structure of the formed ZnO NPs; on the contrary, a significant enhancement in their bioactivity was observed. The possible mechanisms of antioxidant, antibacterial, and anticancer activities were investigated to determine the role of *C. argentea* extract on the bioactivity of ZnO NPs. The biogenic synthesis of ZnO NPs can contribute to modulating the scavenging ability due to phytochemicals such as phenolics and polyphenolic compounds in the plant extract. The physicochemical properties such as size, shape, and surface chemistry, along with the high surface-to-volume ratio, are the main responsible parameters for the antibacterial action of biogenic ZnO NPs on different bacterial strains. The anticancer property of biogenic ZnO NPs is due to the generation of ROS, which mediates the protein activity disequilibrium. The oxidative stress generated can lead to necrosis or DNA damage. As a result of DNA damage, the mitochondrial apoptotic (p53 gene) pathway will be activated, causing cell death by apoptosis. Finally, globally, the biogenic synthesized ZnO NPs *C. argentea* extract could satisfy the new generation of antioxidant, antibacterial, and anticancer nano-drugs. Further investigation is still needed on the *in vivo* behavior of the NPs.

## Data Availability

The datasets presented in this study can be found in online repositories. The names of the repository/repositories and accession number(s) can be found in the article/Supplementary Material.
